# Supplementary material for: Programmable material via thiol-ene polymerization initiated by electric-field induced thiyl radical on piezoelectric ZnO
Source: Nat Commun. 2025 Oct 9;16:8987. doi: 10.1038/s41467-025-64011-y (PMC12511436; doi:10.1038/s41467-025-64011-y)
Supplement: Supplementary file 1 — Supplementary Information [file 41467_2025_64011_MOESM1_ESM.pdf]

Supplementary Materials for

Programmable Material via Thiol-ene Polymerization Initiated by Electric-field Induced Thiyl Radical on Piezoelectric ZnO

Jun Wang[1†], Zhao Wang[2†], Jorge Ayarza[1], Ian Frankel[3], Chao-Wei Huang[1], Kai Qian[3], Yixiao Dong[1], Pin-Ruei Huang[1], Katie Kloska[1], Chao Zhang[1], Siqi Zou[1], Matthew Mason[4], Chong Liu[1], Nicholas Boechler[3], Aaron P. Esser-Kahn[1*]

[†]These authors contribute equally to this work.
[1]Pritzker School of Molecular Engineering, University of Chicago, Chicago, IL 60637, USA

[2]College of Chemistry, Chemical Engineering and Materials Science, Soochow University, Suzhou 215123, China

[3]Department of Mechanical and Aerospace Engineering, University of California San Diego, San Diego, CA 92121, USA

[4]Department of Chemistry, Princeton University, Princeton, NJ 08540, USA

*Correspondence to: aesserkahn@uchicago.edu.

1. Supplementary figures

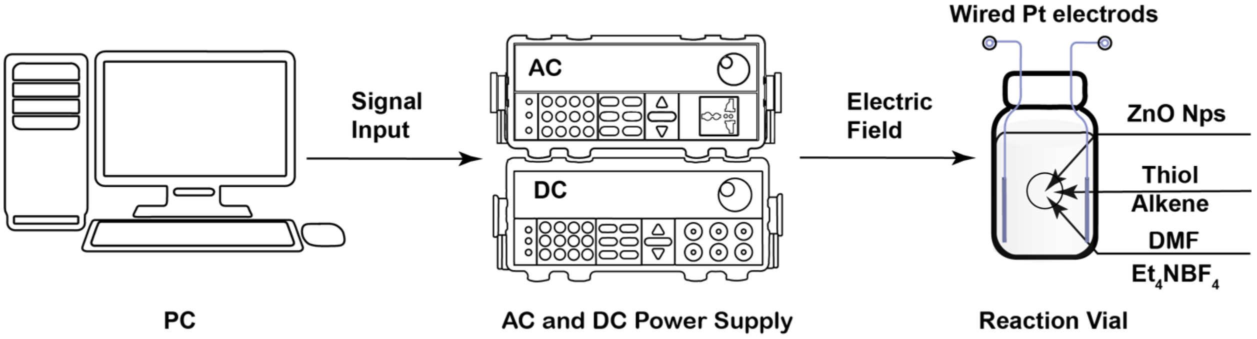

Supplementary Figure 1. Schematic diagram of the experimental setup for electric field-induced thiol-ene system.

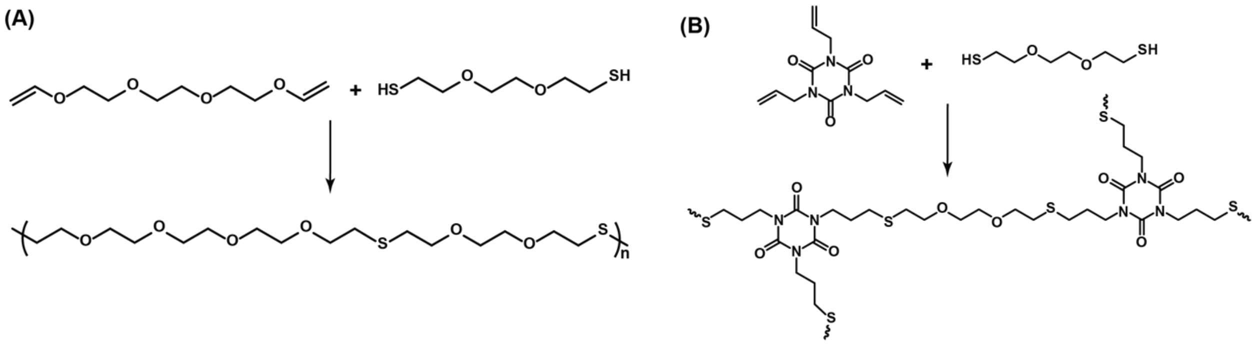

Supplementary Figure 2. (A) Linear polymerization between TEGDE and EDT; (B) Crosslinking reaction between TTT and EDT.

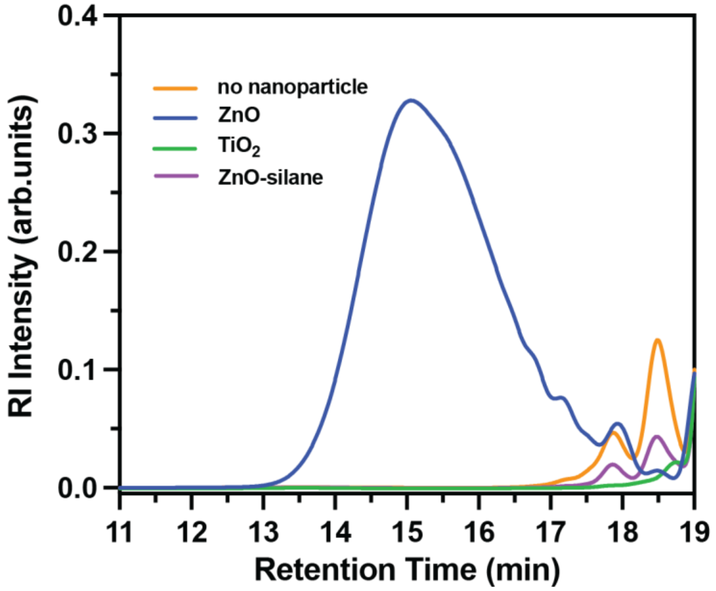

Supplementary Figure 3. GPC traces of thiol-ene linear polymerization conducted with and without selected nanoparticles under AC electric field (500 Hz, 8 V_{rms}, 3 h).

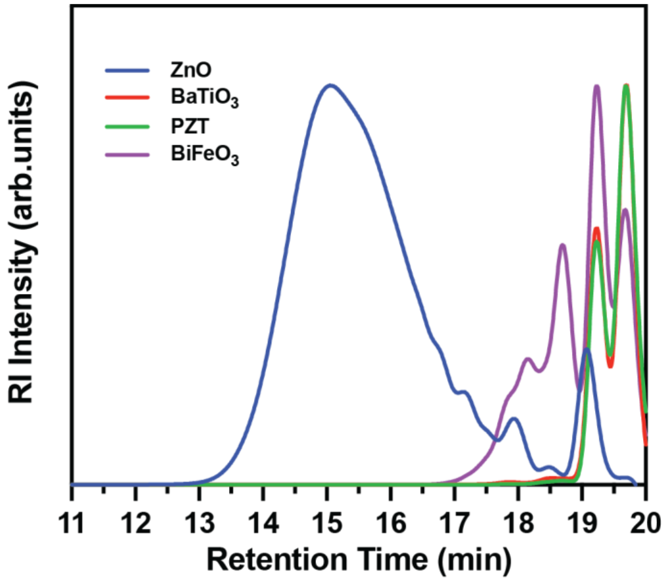

Supplementary Figure 4. GPC traces of thiol-ene linear polymerization conducted with different piezoelectric nanoparticles under AC electric field (500 Hz, 8 V_{rms}, 3 h).

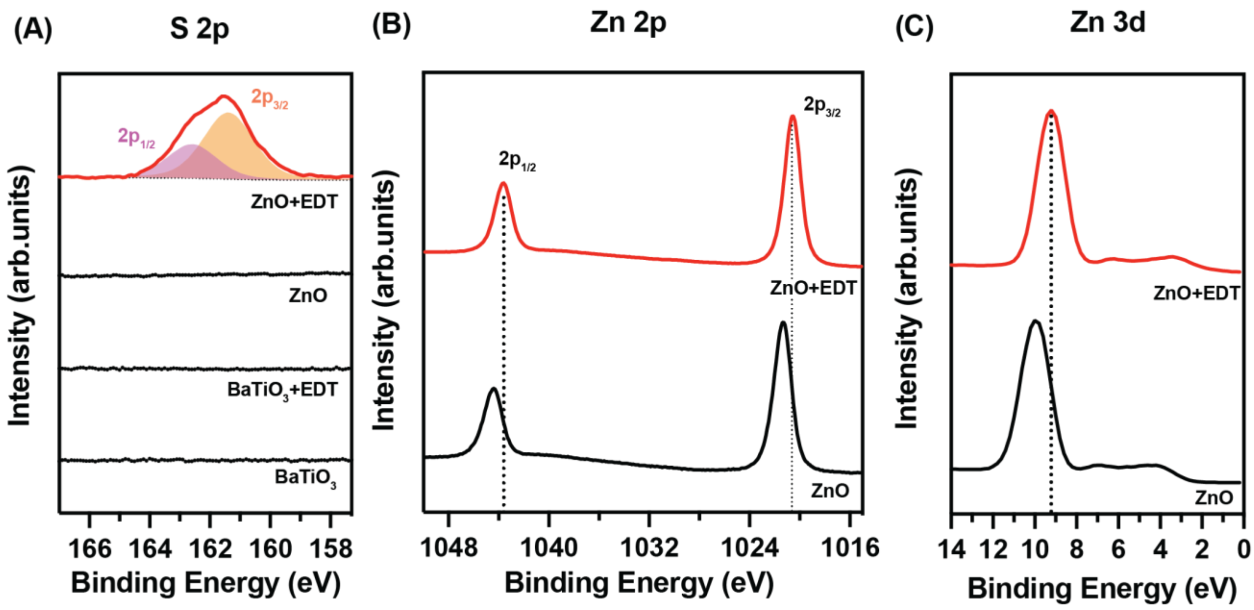

Supplementary Figure 5. High-resolution XPS spectra of (A) S 2p, (B) Zn $2p_{3/2}$ and (C) Zn 3d regions recorded with the pure and EDT treaded nanoparticle samples.

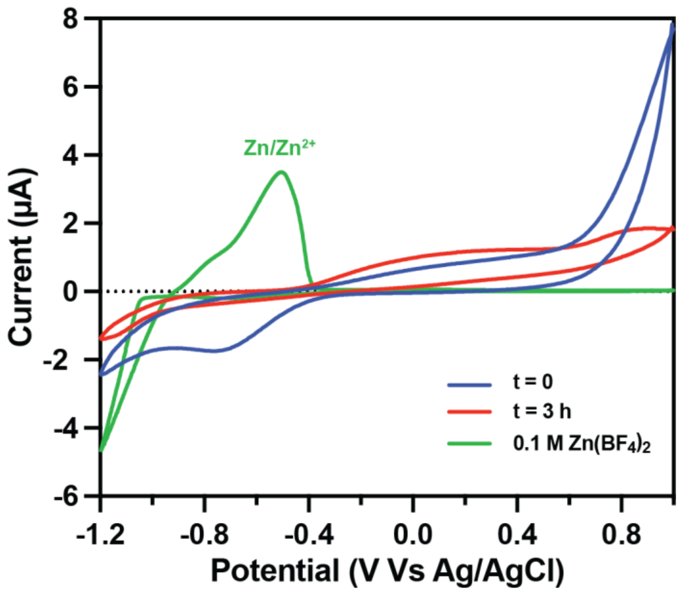

Supplementary Figure 6. Cyclic voltammetry curves for the thiol-ene linear polymerization mixture supernatant before (blue line) and after (red line) undergoing AC electric field (8 V_{rms}, 500 Hz, 3h). The green line represents the CV curve obtained with 0.1 M $Zn(BF_4)_2$ in DMF.

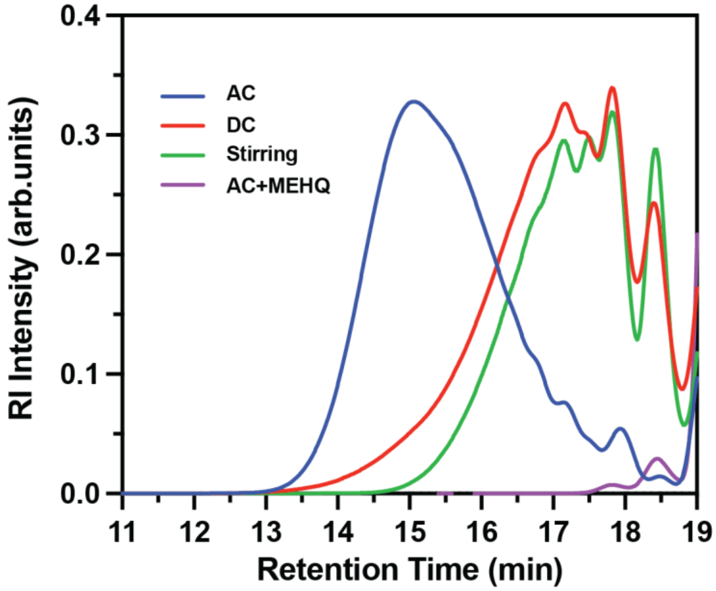

Supplementary Figure 7. GPC traces of thiol-ene linear polymerization conducted under AC electric field (8 V_{rms}, 500 Hz), DC electric field (8 V) and stirring (500 rpm) conditions for 3 hours.

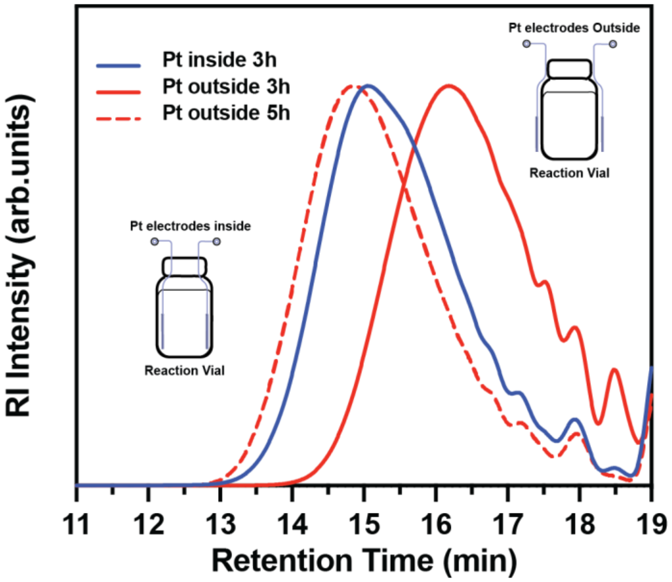

Supplementary Figure 8. GPC traces of thiol-ene linear polymerization conducted with Pt electrodes inside and outside of the reaction vials under AC electric field (8 V_{rms}, 500 Hz).

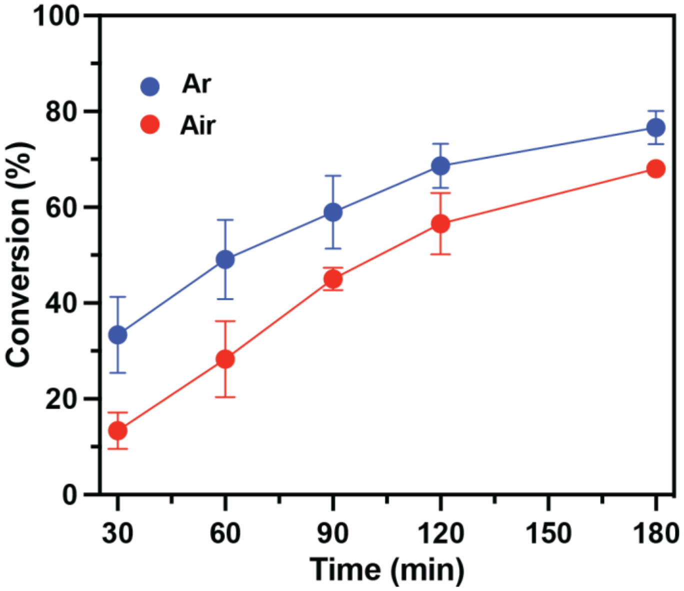

Supplementary Figure 9. Kinetics of thiol-ene linear polymerization conducted with AC electric field (8 V_{rms}, 500 Hz) for 30 min under Ar and Air. All the error bars are SD calculated with results of three independent experiments.

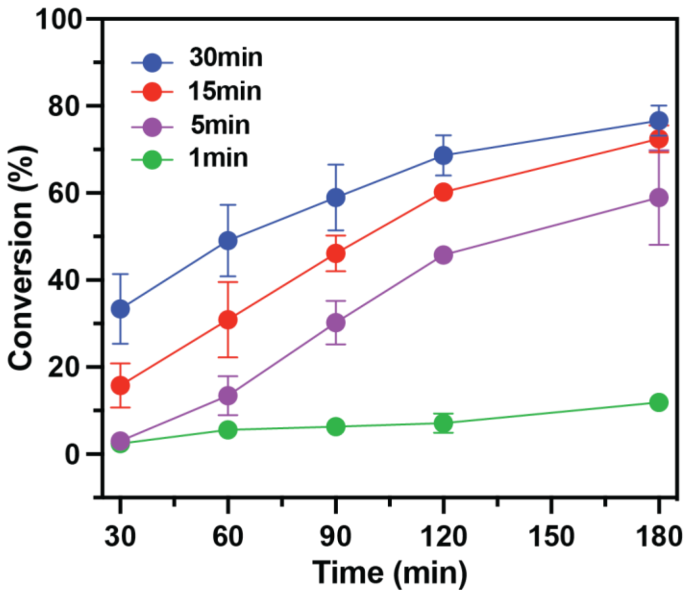

Supplementary Figure 10. Kinetics of thiol-ene linear polymerization conducted in glove box under AC electric field (8 V_{rms}, 500 Hz) for various times (1-, 5-, 15- and 30- min). The x-axis denotes the time over which the reaction was monitored (wherein, e.g. in the case of 1-minute plot (green line), the electric field was applied for 1 minute and then left off for 29 minutes, after which the conversion measurement began). All the error bars are SD calculated with results of three independent experiments.

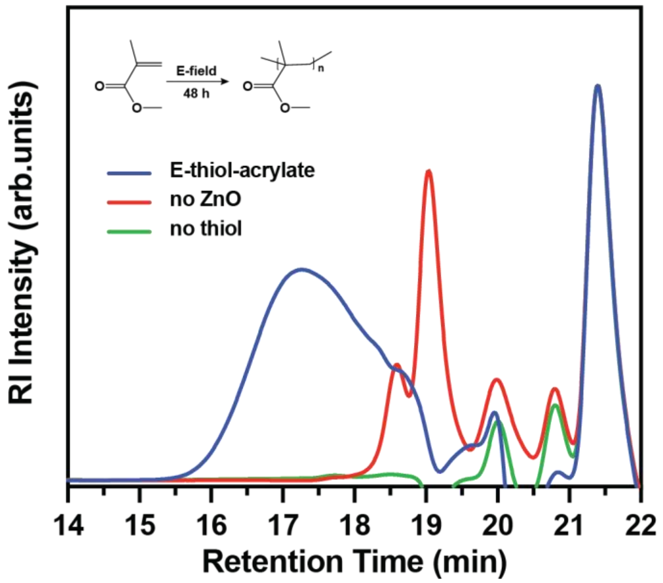

Supplementary Figure 11. GPC traces of acrylate-based polymerization linear polymerization conducted with Pt electrodes outside of the reaction vials under AC electric field (50 V_{rms}, 500 Hz, 48 h).

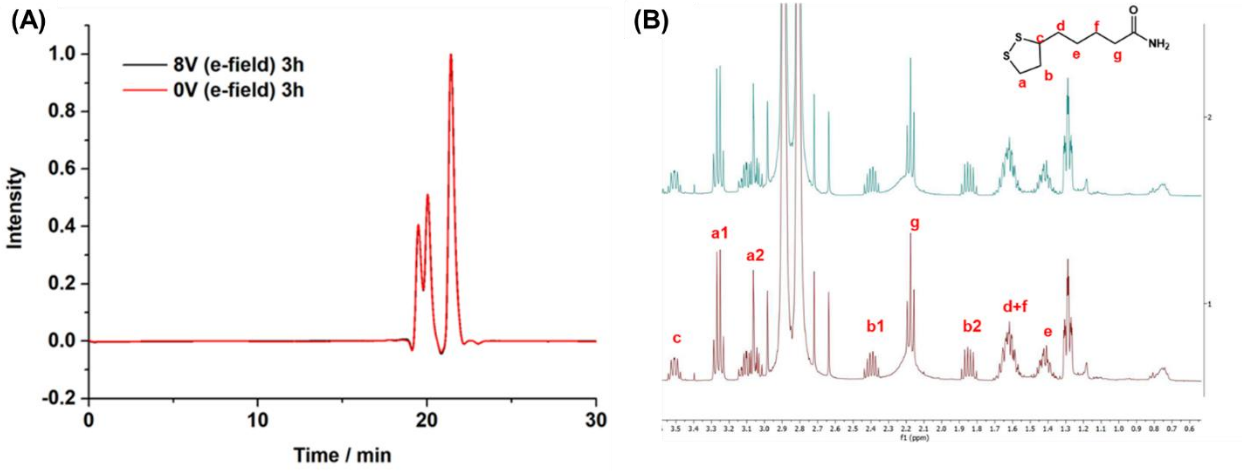

Supplementary Figure 12. (A) GPC traces of disulfide polymerization before and after AC electric field (8 V_{rms}, 500 Hz, 3h). (B) NMR results also show that no polymer was produced since no monomer peak is reduced and potential new polymer peaks are not found. (top NMR plot: before E-field stimulation; bottom NMR plot: 3 h after E-field stimulation)

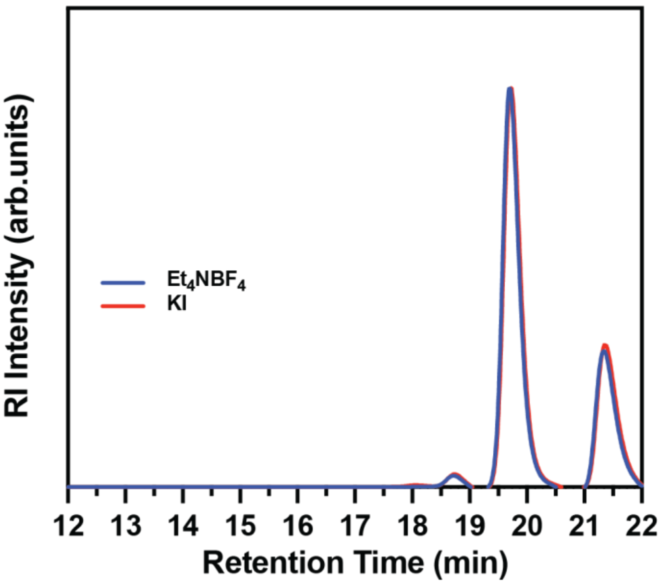

Supplementary Figure 13. GPC traces of E-disulfide polymerization under AC electric field (8 V_{rms}, 500 Hz, 3h) using KI and Et_4NBF_4 as supporting electrolytes.

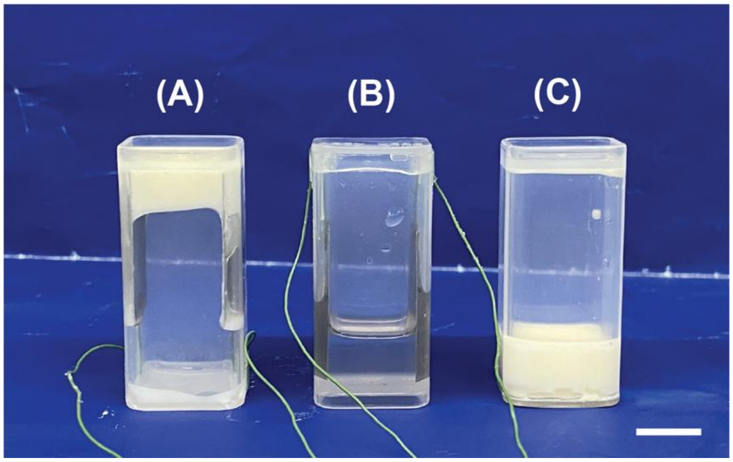

Supplementary Figure 14. Photographs of thiol-ene crosslinking reaction solution. (A) Solution containing ZnO under AC electric field (500 Hz, 8 V_{rms}, 3 h); (B) Solution without ZnO under AC electric field (500 Hz, 8 V_{rms}, 3 h) and (C) Solution containing ZnO without electric field. Scale bar: 10 mm.

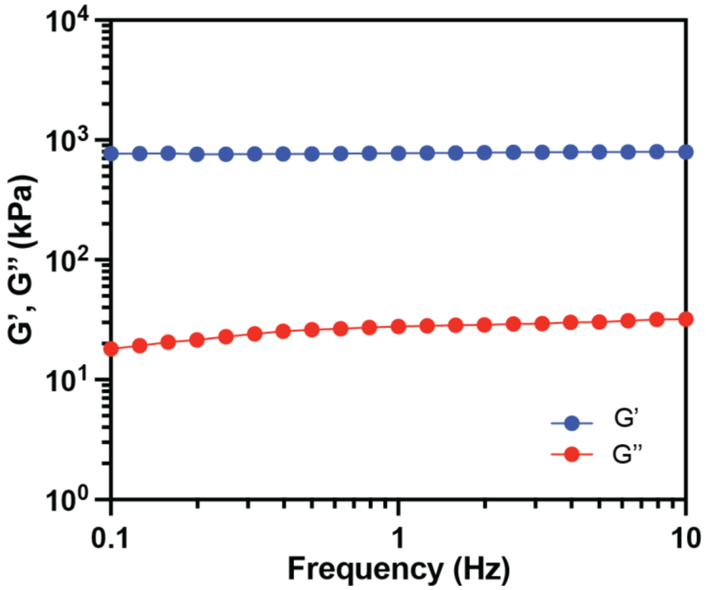

Supplementary Figure 15. Storage (G') and loss (G") moduli as a function of frequency (Hz) of the thiol-ene gel in Fig. S 14A. Measurements were conducted at a fixed shear strain amplitude of 1%.

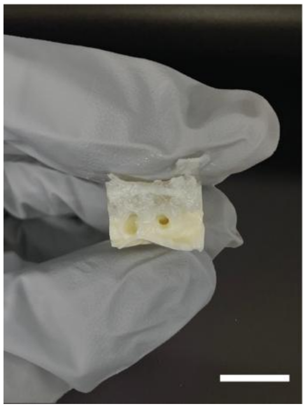

Supplementary Figure 16. Photograph of thiol-ene crosslinking gel sample obtained after DC electric field (8V, 3h). Scale bar: 10 mm.

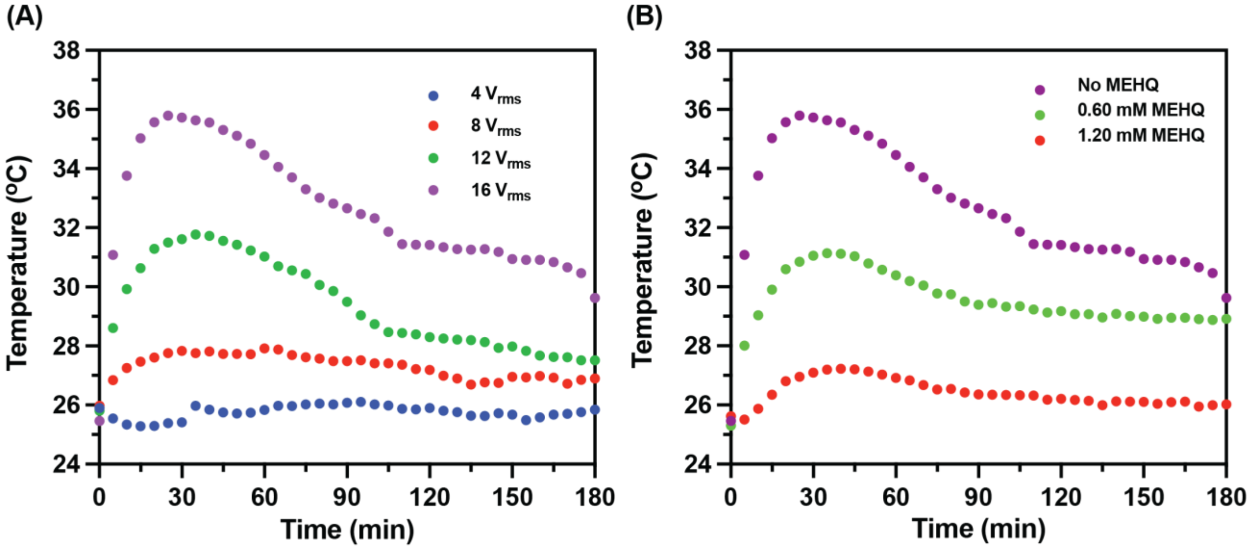

Supplementary Figure 17. Time-dependent temperature changed plots for thiol-ene crosslinking gelation conducted (A) under various AC electric fields and (B) with various concentration of MEHQ under AC voltage (16 V_{rms}, 500 Hz)

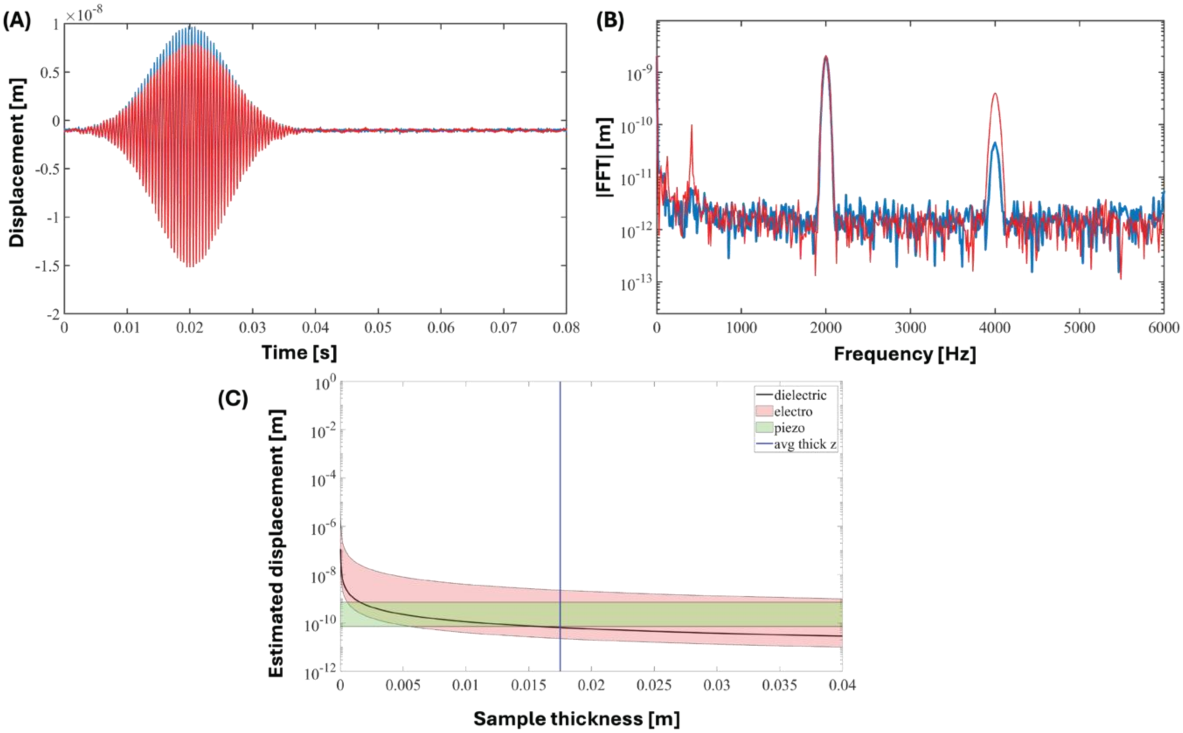

Supplementary Figure 18. (A) Displacement of the 1.70 cm thick control sample with TiO_2 nanoparticles (blue) and the 1.80 cm thick ZnO nanoparticle sample (red) excited with a 200 V_{pp} 2000 Hz sine-modulated Gaussian wave with a 100V DC offset. (B) Fourier transform of (A) showing the control TiO_2 sample in blue, and the ZnO sample in red. (C) Expected displacement contribution vs. thickness for a ZnO thiol-ene sample with the upper and lower bound electrostriction shaded in red, dielectric elastomer effect in black, and upper and lower bound piezoelectric effect shaded in green with the average thickness of the samples (1.75 cm) plotted in purple.

In Supplementary Fig. 18, the electrostrictive displacement was obtained via the relationship $\delta_e = ME^2/z$, where M is the electrostrictive coupling value, the M value was approximated from values of polyvinylidene fluoride (PVDF) and polyurethane (PU) polymers (10^{-15} to 10^{-17} m^2/V^2)[1] and E is the electric field, for our setup of two narrowly spaced parallel plates is given by $E = U/z$. The displacement from dielectric elastomer effect was calculated via the stress generated in the material[2] $\sigma = \varepsilon_0\varepsilon_r U^2/z^2$, where ε_0 is the permittivity of free space (8.85×10^{-10} m^{-3} kg^{-1} s^4 A^2), ε_r is the dielectric constant of the material (5.31) which was measured on a Discovery Hybrid Rheometer, U is the excitation voltage, and z is the material thickness. The strain can then be obtained from Hooke's Law, where $\sigma = E_y\varepsilon_d$, where E_y is the Young's modulus of the testing material, which was experimentally measured to be 1.655 MPa, and E_y is the strain in the material. The displacement is then given by $\delta_d = \varepsilon_d z$. The estimated displacement from the converse piezoelectric effect is linearly related to the electric field from the piezoelectric constant d_{33}**.** An upper bound estimate of the piezoelectric constant $d_{33} = 3.6 \times 10^{-12}$ C/N which is the piezoelectric constant for ZnO microtubes[3]. The lower bound estimate for piezoelectric constant was taken as 3.6×10^{-13} C/N or 10 % of the upper bound d_{33}. In reality, the d_{33} of composite sample is most likely around an order of magnitude smaller and our estimation of dielectric displacement is most

likely larger than reality. The piezoelectric displacement δ is given by $\delta = d_{33}U$, where U is applied voltage.

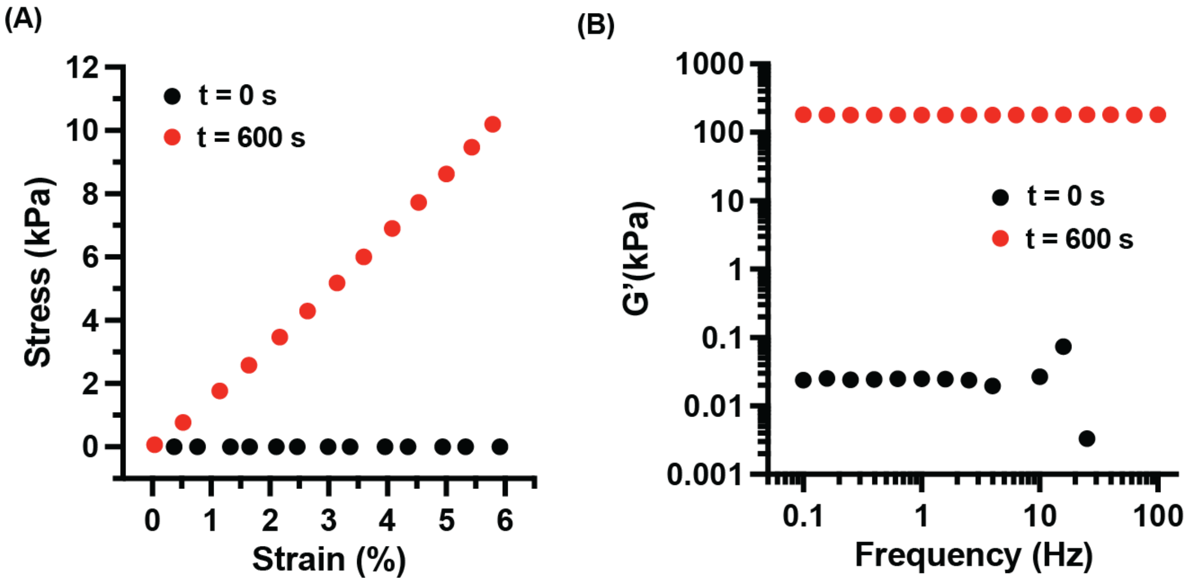

Supplementary Figure 19. (A) Strain-stress curves and (B) Storage modulus (G')[a] as a function of frequency (Hz) of the thiol-ene samples in Figure 3b (i) and Figure. 3b (ii). [a]Measurements were conducted at a fixed shear strain amplitude of 1%.

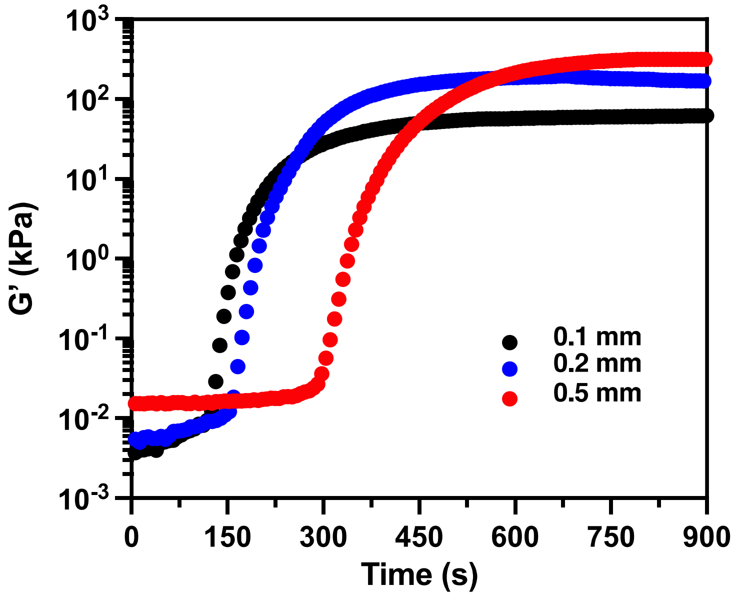

Supplementary Figure 20. Time-dependent evaluation of storage modulus (G′) with various gap distance under AC voltage (2 V_{rms}, 2k Hz).

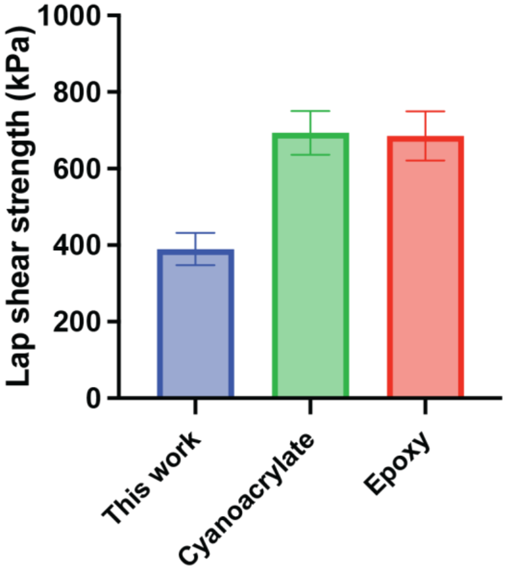

Supplementary Figure 21. Lap shear strength of electro-adhesive (this work) and commercial adhesive samples on ITO-coated glass substrates at 25 °C. All the error bars are SD calculated with results of three independent experiments.

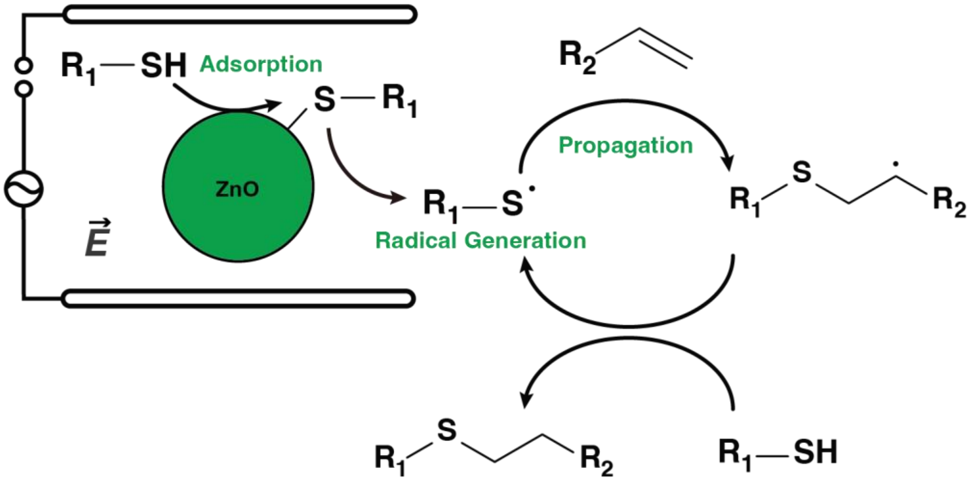

Supplementary Figure 22. The plausible reaction mechanism of thiol-ene reaction under electric field.

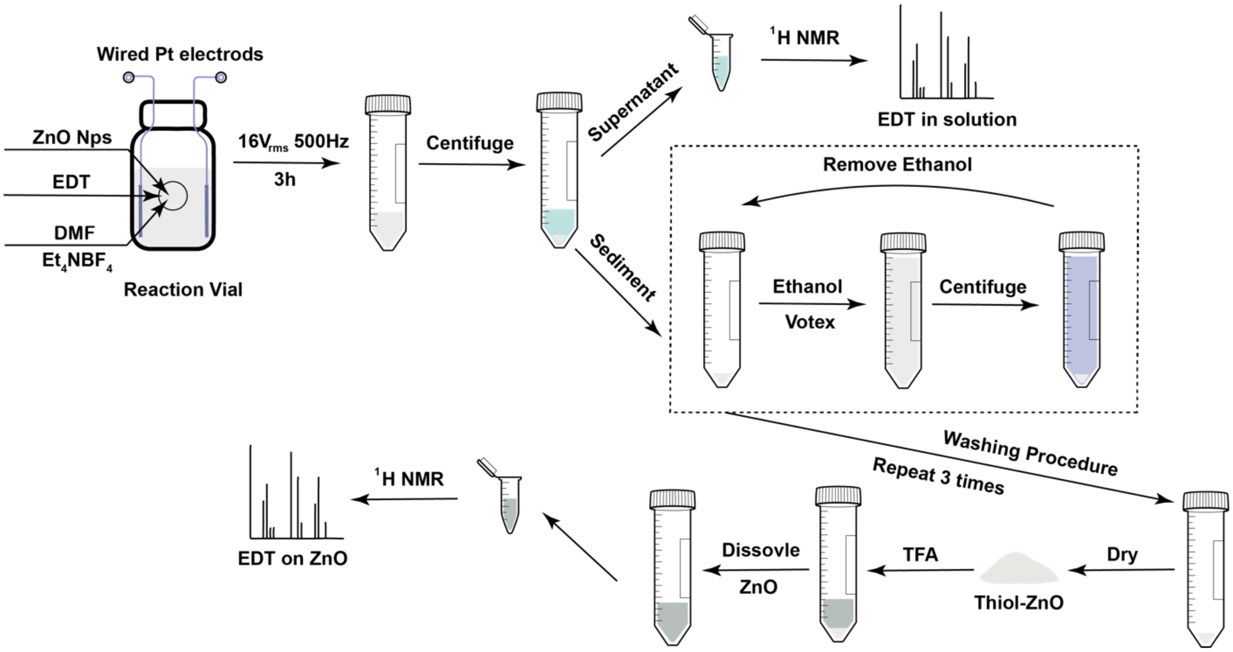

Supplementary Figure 23. Workflow diagram for the quantitative evaluation of EDT adsorption on the surface of ZnO nanoparticles.

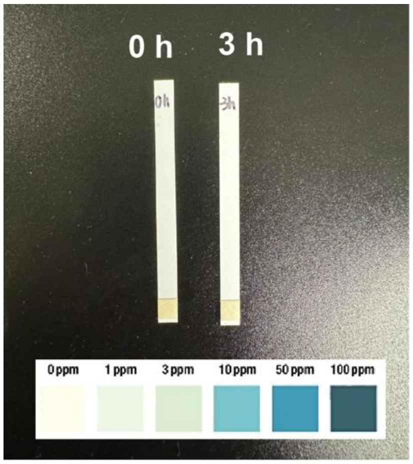

Supplementary Figure 24. Photographs of hydrogen peroxide testing strips before (0 h, left) and after (3 h, right) thiol-ene reaction.

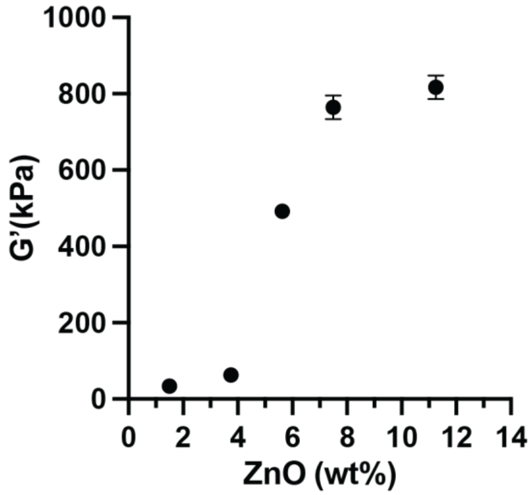

Supplementary Figure 25. Storage modulus of the thiol-ene gel as a function of ZnO concentration (1.50 to 11.25 wt%) under AC voltage (16 Vrms, 500 Hz, 3 h). Measurements were performed at a fixed strain amplitude of 1%. The error bars represents the SD of three independent experiment results.

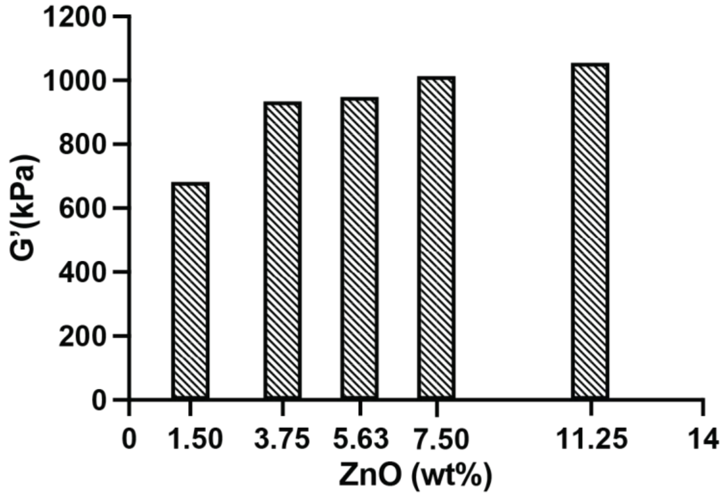

Supplementary Figure 26. Storage modulus of the thiol-ene gel as a function of ZnO concentration (1.50 to 11.25 wt%) under heat (100 °C, 24 h) until fully crosslinked. Measurements were performed at a fixed strain amplitude of 1%.

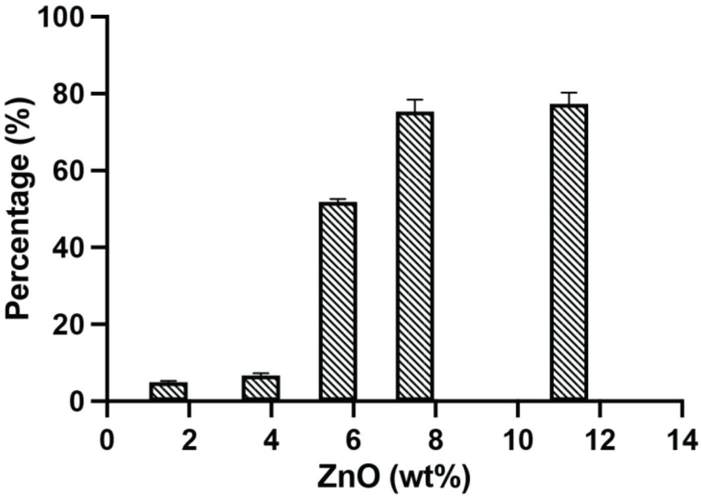

Supplementary Figure 27. Comparison of storage modulus (G′) of thiol-ene gels formed by AC field–induced gelation (16 V_{rms}, 500 Hz, 3 h). and thermal curing (100 °C, 24 h) as a function of ZnO concentration (1.50 to 11.25 wt%). Measurements were performed at a fixed strain amplitude of 1%. The error bars represent the SD of three independent experiment results.

2. Supplementary tables

Supplementary Table 1. Summary of thiol-ene linear polymerization reactions between TEGDE and EDT for 3 hours.

Entry	ZnO	Condition	Conversion (%)[a]	M_n (kDa)[b]
1	Yes	AC (8 $V_{rms,}$ 500 Hz)	90.20	4.2
2	Yes	DC (8 V)	65.25	3.0
3	Yes	Stirring (500 rpm)	56.25	2.0
4	No	AC (8 $V_{rms,}$ 500 Hz)	6.19	0.6
5	Yes	AC (8 $V_{rms,}$ 500 Hz); 0.6 mmol MEHQ	4.49	0.5

[a] The conversion was determined by ^{1}H NMR spectroscopy.

[b] The M_n was determined by GPC-MALLS in THF.

Supplementary Table 2. The zeta potential of ZnO nanoparticles under different DC electric fields.

DC Voltage (V)	Zeta potential (mV)
5	-23.0
10	-22.3
20	-20.9
50	-18.1

Supplementary Table 3. Results of thiol-ene linear radical polymerization inhibited by 4-methoxyphenol (MEHQ) under AC electric field (4 V_{rms}, 500 Hz) for 3 h.

Entry	AC	MEHQ concentration (mmol)	Conversion (%)[a]
1	4 V_{rms}, 500 Hz	0.15	68.7
2	4 V_{rms}, 500 Hz	0.30	7.08
3	4 V_{rms}, 500 Hz	0.45	2.69
4	4 V_{rms}, 500 Hz	0.60	0.45

[a] The conversion was determined by ^1H NMR spectroscopy.

Supplementary Table 4. Results of thiol-ene linear radical polymerization inhibited by 4-methoxyphenol (MEHQ) under various AC electric fields (500 Hz, 3 h).

Entry	AC	MEHQ concentration (mmol)	Conversion (%)[a]
1	4 V_{rms}, 500 Hz	0.30	7.08
2	8 V_{rms}, 500 Hz	0.30	44.1
3	12 V_{rms}, 500 Hz	0.30	71.3
4	16 V_{rms}, 500 Hz	0.30	77.9

[a] The conversion ratio was determined by ^1H NMR spectroscopy.

Supplementary Table 5. Results of EDT (3 mmol) adsorption on the surface of ZnO nanoparticles (210 mg).

Thiol-ZnO (mg)	EDT in solution (mmol)	EDT on ZnO (mmol)	EDT lost in washing (mmol)
258.9±1.2	2.644±0.135	0.197±0.002	0.1387±0.013

Supplementary Table 6. Results of conversion of tri(ethylene glycol) divinyl ether (TEGDE) react with thiol-ZnO under AC voltage (16 V_{rms}, 500 Hz, 3 h).

Entry	AC	Thiol-ZnO (mg)	EDT (mmol)	Conversion (%)[a]
1	16 V_{rms}, 500 Hz	258.18	0	16.5
2	No AC	259.35	0	0.15

[a] The conversion was determined by ^1H NMR spectroscopy.

Supplementary Table 7. Energy efficiency comparison between e-field curing system (this work) and the existing light curing in literatures.

Entry	Power source	Power intensity	Curing time	Ref
1	AC generator	58 mW/cm^2	~10 min (without initiator)	This work
2	Uvitron UV 1080 Flood Curing System	120 mW/cm^2	<1min (with inititator)	4
3	EFOS Acticure (mercury vapor lamp)	15 mW/cm^2	15-30 min (without initiator)	5

3. Supplementary references

1 Lallart, M., Cottinet, P., Guyomar, D. & Lebrun, L. Electrostrictive polymers for mechanical energy harvesting. *J. Polym. Sci. Part B Polym. Phys.* **50**, 523–535 (2012).

2 Onyenucheya, B., Allen, J., Pierre, K., Zirnheld, J. & Burke, K. Dielectric Elastomers: An Investigation in Strain Dependent Electrostatic Pressure of Soft Compliant Dielectric. in *2019 IEEE Pulsed Power & Plasma Science (PPPS)* 1–4 (IEEE, Orlando, FL, USA, 2019). doi:10.1109/PPPS34859.2019.9009996.

3 Fu, J. Y., Liu, P. Y., Cheng, J., Bhalla, A. S. & Guo, R. Optical measurement of the converse piezoelectric d33 coefficients of bulk and microtubular zinc oxide crystals. *Appl. Phys. Lett.* **90**, 212907 (2007).

4 Mautner, A., Qin, X., Wutzel, H., Ligon, S. C., Kapeller, B., Moser, D., ... & Liska, R.. Thiol-ene photopolymerization for efficient curing of vinyl esters. *Journal of Polymer Science Part A: Polymer Chemistry*, **51**, 203-212 (2013)

5 Cramer, N. B., Scott, J. P., & Bowman, C. N. Photopolymerizations of thiol− ene polymers without photoinitiators. *Macromolecules*, **35,** 5361-5365 (2002).
