# Supplementary material for: Programmable material via thiol-ene polymerization initiated by electric-field induced thiyl radical on piezoelectric ZnO
Source: Nat Commun. 2025 Oct 9;16:8987. doi: 10.1038/s41467-025-64011-y (PMC12511436; doi:10.1038/s41467-025-64011-y)
Supplement: Supplementary file 2 — Description of Additional Supplementary Files [file 41467_2025_64011_MOESM2_ESM.pdf]

Description of Additional Supplementary Files

File Name: Supplementary Movie 1
Description: demo for the control adhesive sample without electricity.

File Name: Supplementary Movie 2
Description: demo for the activation of Eadhesive sample via 3V-battery.
